# Menstrual problems and associated factors among students of Bahir Dar University, Amhara National Regional State, Ethiopia: A cross-sectional survey

**DOI:** 10.11604/pamj.2014.17.246.2230

**Published:** 2014-04-01

**Authors:** Muluken Teshome Shiferaw, Mamo Wubshet, Desalegn Tegabu

**Affiliations:** 1 Department of Public Health, Debre Markos University, PO Box-269, Debre Markos, Ethiopia; 2 Department of Environmental Health, University of Gondar, PO Box-196, Gondar, Ethiopia; 3 Department of Epidemiology and Biostatistics, University of Gondar, PO Box-196, Gondar, Ethiopia

**Keywords:** Dysmenorrhea, menstruation, premenstrual syndrome

## Abstract

**Introduction:**

Menstrual problems are the most common gynecologic complaints. The prevalence is highest in the 20 to 24-year-old age group and decreases progressively thereafter. They affect not only the woman, but also family, social and national economics as well. However, Population studies on Menstrual problems and associated factors were very little for university students in Ethiopia.

**Methods:**

Institutional based quantitative cross-sectional study was employed at Bahir Dar University from October 14 to 20, 2010, Ethiopia. Stratified sampling technique was used and 491 study subjects were randomly selected from faculties. Only 470 respondents had given complete response for the self-administered questionnaire and were included in the final analysis. Data was entered and analyzed with SPSS version 16.0 windows. The main statistical method applied was logistic regression (unconditional) and both the classical bivariate and the multivariate analyses were considered.

**Results:**

The prevalence of dysmenorrhea and premenstrual syndrome were 85.1% and 72.8%, respectively. The most contributing factors remained to be statistically significant and independently associated with dysmenorrhea were having menstrual cycle length of 21-35 days (AOR=0.16, 95%CI: 0.04, 0.71), family history of dysmenorrhea (AOR=3.80, 95%CI: 2.13, 6.78) and circumcision (AOR=1.84, 95%CI: 1.001, 3.386) while with premenstrual syndrome were educational status of mothers being certified in certificate and beyond (AOR=0.45, 95%CI: 0.25, 0.83), living in Peda campus (AOR=2.11, 95%: 1.30, 3.45), having irregular menstruation (AOR=1.87, 95%CI: 1.17, 2.99) and family history of premenstrual syndrome (AOR=4.19, 95%CI: 2.60, 6.74).

**Conclusion:**

The prevalence of menstrual problems among students of Bahir Dar University was very high. Menstrual cycle length, family history of dysmenorrhea and circumcision were the most contributing factors associated with dysmenorrhea while educational status of mothers, regularity of menstruation, and family history of premenstrual syndrome were for premenstrual syndrome. Health education, appropriate medical treatment and counseling, should be accessible and persistently provided to the affected students by Bahir Dar University. Maximum effort is needed to eliminate circumcision by all levels and further steps that would enable females to join their college education should be applied.

## Introduction

Menstruation is the periodic change occurring in primates, which results in the flow of blood and endometrium from the uterine cavity, and which may be associated with various constitutional disturbances. Dysmenorrhea which is one of the most common gynecologic complaints in young women who present to clinicians can be defined as difficult menstrual flow or painful menstruation [1–3]. Premenstrual syndrome (PMS) is the name given to a collection of physical and psychological symptoms that some women experience during the late luteal phase of each menstrual cycle (7 to 14 days prior to menstruation). Although various etiologies of premenstrual syndrome such as elevated prolactin levels, hypoglycemia or vitamin deficiencies have been proposed, none of these theories has been definitively proven [4, 5].

The prevalence of primary dysmenorrhea decreases with increasing age; prevalence is highest in the 20 to 24 year old age group and decreases progressively thereafter. On the other hand, premenstrual syndrome is a multifactorial syndrome that affects adolescent girls with a high frequency. It affects millions of women during their reproductive years. Both dysmenorrhea (usually of the primary type) and PMS are common problems and have negative effect on a woman's life [6–9]. Making the diagnosis of PMS has been problematic, since its specific etiology is unknown and there is no objective marker which can quantitate the existence or the severity of symptomatology or even the objective response to therapy. The diagnostic and statistical manual of mental disorders (DSM-IV) classified PMS as a mental disorder and termed it the premenstrual dysphoric disorder (PMDD). Our study used the diagnostic criteria proposed by DSM-IV to diagnose PMS [10].

Menstrual problems affect not only the woman, but also family, social and national economics as well. However, Population studies on menstrual problems and associated factors are very little for adolescent female university students in Ethiopia. Therefore, this study will come up with the magnitude of menstrual problems and associated factors at the university level.

## Methods

**Study design:** Institutional based quantitative cross-sectional study was employed.

**Study area and Period:** The study was conducted from October 14 to 20, 2010 in Bahir Dar University (BDU) which is one of the well known universities in Ethiopia. BDU is found in Bahir Dar town which is the capital city of Amhara National Regional State (ANRS) and located at 565 km North West of Addis Ababa, capital city of Ethiopia.

**Sampling:** Sample size was calculated by assuming 95% confidence level, 4% absolute precision or marginal error, 72% prevalence of dysmenorrheal which was found from a study of secondary school adolescents of Northern Ethiopia. Assuming non-response rate of 10%, the total sample size was 491. Any regular undergraduate female student in the academic year 2009/2010 who had seen her menarche had the chance to be included in the study. Stratified sampling technique was used. The total sample was allocated according to proportion to population size of both campuses (Poly and Peda), year of study and faculties. Then, the study subjects were selected by simple random sampling technique from each faculty.

**Data collection:** A pre-tested, structured and self administered questionnaire was used to collect the data. It was prepared in English then translated to Amharic and again back to English. Questions in relation to socio- demography, Environmental/ behavioral, Obstetric/Gynecologic information were included.

Data was collected by distributing the pretested, structured Amharic version questionnaire to each respondent after we got oral consent and voluntariness from each respondent. Explanation on the objective of the study, relevance of the study and how to fill the questionnaire to the study subjects was given before they filled the questionnaire. It was collected from October 14 to 20 2010. Besides the principal investigator, five female nurses (data collectors) and two Master of Public Health students (supervisors) had participated throughout the data collection process.

**Data Analysis:** The data errors related to inconsistency were checked and corrected during data cleaning. Data was entered and analyzed using SPSS version 16.0 windows. The univariate analyses (proportions, percentages, and ratios) had been displayed. The logistic regression (unconditional), in both the classical bivariate analyses and multivariate analysis, was considered. The technique was backward stepwise regression. The unadjusted (crude) and adjusted odds ratios together with their corresponding 95% confidence interval had been computed. A p-value ≥ 0.05 was considered statistically significant in this study. Efforts were made to assess whether the necessary assumptions for the application of multiple logistic regression were fulfilled. For this, the Hosmer and Lemeshow's goodness-of-fit test was considered. A good-fit as measured by Hosmer and Lemeshow's test will yield a large P-value. Ethical clearance was obtained from the Institutional Review Board of the College of Medicine and Health Sciences of the University of Gondar. Moreover, respondents were briefed about the objectives, disadvantages and relevance of the study. Participants’ privacy and confidentiality of the information was maintained by using anonymous type of self administered data collection tool. Verbal consent was obtained from each participant to ensure their voluntariness to participate in the study. Respondents had given the right to put an end for the question or segment of questions or refuse to participate at all. After data collection, health education on the normal physiology of menstruation and menstrual problems was given for the study subjects by the investigator.

## Results

**Figure 1:**
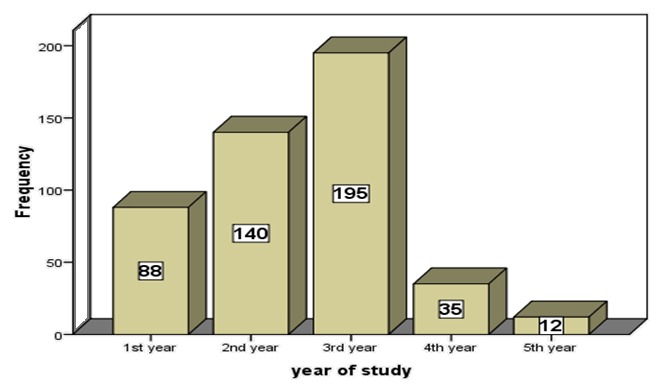
Number of students by year of study, BDU, ANRS, Ethiopia, 2010

**Figure 2:**
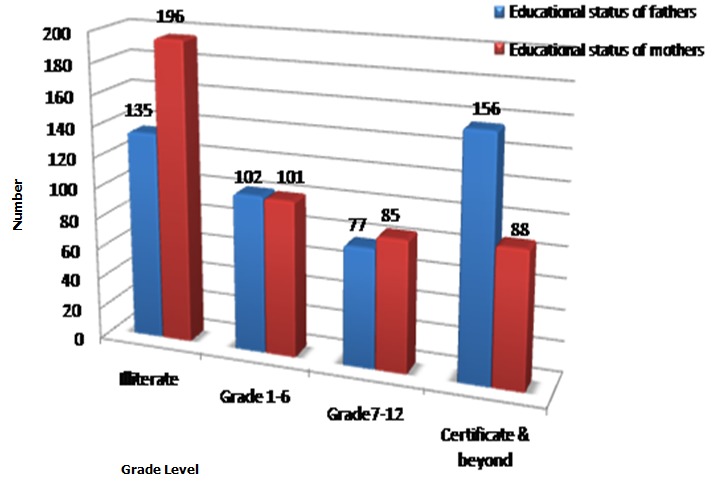
Educational status of parents of the study subjects, BDU, ANRS, Ethiopia, 2010

From the total 491 study subjects, only 470 had completed the questionnaire. This makes the response rate 95.7%. The age of respondents ranges between 17 to 24 years with the mean age of 20.4±1.2 years. Most (82.7%) of the respondents were Orthodox in religion. Most (92.3%) of them were single in marital status. Majority (70.7%) of the respondents were Amhara in ethnicity. About 26.2% and 25.3% of the respondents came from Woreda Town and Addis Ababa, respectively. The educational status of fathers was majority (33.2%) certificate and above while that of mothers was majority (41.7%) illiterate. Most (71.5%) of the proportionally allocated respondents were from Peda campus. About 29.4% of them were in social science faculty and the majority (41.5%) were 3^rd^ year students (Table 1, Figure 1, Figure 2).

**Table 1:** Socio-demographic characteristics of the students of BDU, ANRS, Ethiopia, 2010

Characteristics	Frequency (N=470)	Percent (%)
Age (year)		
15-19	99	21.1
20-24	371	78.9
**Marital Status**		
Single	434	92.3
Married	32	6.8
Divorced	4	0.9
**Religion**		
Orthodox	389	82.7
Muslim	38	8.1
Protestant	38	8.1
Others	5	1.1
**BMI (kg/m** ^**2**^ **)**		
Under weight (BMI <18.50)	131	27.9
Normal BMI 18.50-24.99)	307	65.3
Over weight (BMI >24.99	32	6.8
**Ethnicity**		
Amhara	332	70.7
Tigre	46	9.8
Oromo	55	11.7
Gurage	27	5.7
Others	10	2.1
**Residence before joining university**		
Addis Ababa	119	25.3
Regional town	81	17.2
Zonal town	57	12.1
Woreda town	123	26.2
Rural	90	19.2
**Father's occupation**		
Farmer	170	36.2
Merchant	94	20.0
Private employee	61	12.9
Government employee	145	30.9
**Mother's occupation**		
Housewife	305	64.9
Merchant	70	14.9
Private employee	16	3.4
Government employee	79	16.8
**Campus**		
Poly	134	28.5
Peda	336	71.5
**Faculty**		
Social science	138	29.4
Natural science	76	16.2
Business and Economics	120	25.5
Agriculture	31	6.6
Engineering	101	21.5
Medicine	4	0.8
**Family monthly income**		
≤ 1000	154	32.8
1001-2000	110	23.4
2001-3999	84	17.9
≥ 4000	122	25.9

The age at menarche ranged between 10 and 19 years with mean of 14.7±1.6 years. Menstruation was irregular in 46.2% of the respondents. This study revealed that few (3.8%) of the respondents had menstrual cycle length of shorter than 21 days and the majority (81.3%) of them had between 21 and 35 days, inclusively (Table 2).

**Table 2:** Obstetric/gynecologic characteristics of respondents, BDU, ANRS, Ethiopia, 2010

Characteristics	Frequency (N=470)	Percent (%)
**Menstrual regularity**		
Regular	253	53.8
Irregular	217	46.2
**Menstrual cycle length**		
< 21 days	18	3.8
21-35 days	382	81.3
> 35 days	70	14.9
**Duration of menstrual flow**		
≤ 2 days	50	10.6
3- 7 days	370	78.8
≥ 8 days	50	10.6
**Amount of menstrual flow/cycle**		
Little (≤4 pads/day)	398	84.6
Moderate (5-7 pads/day	52	11.1
Heavy (≥8 pads/day)	20	4.3
**Family history of dysmenorrhea**		
Yes	264	56.2
No	206	43.8
**Family history of PMS**		
Yes	246	52.3
No	224	47.7
**Circumcision**		
Yes	209	44.5
No	261	55.5
**Hormonal family planning**		
Yes	90	19.1
No	380	80.9
**Abortion (ever) (N=134)**		
Yes	33	24.6
No	101	75.4

The overall prevalence of dysmenorrhea (which is assumed to be primary dysmenorrhea since secondary dysmenorrhea is rare at this age) was 85.1%. The number of respondents who had reported PMS (at least one symptom 1-7 days prior to menstruation in the last 12 months) was 342(72.8%) (Table 3). Among these, only 76(22.2%) of them had fulfilled the diagnostic criteria for PMDD. Higher proportion of respondents who had PMS also suffer from dysmenorrhea (93.8%), compared to those who had not PMS (70.3%). The most common five symptoms felt by dysmenorrheic respondents during dysmenorrhea were stomach cramp (72.8%), depression (49.2%), fatigue (47.5%), backache (43.2%), and bloating (41.2%).

**Table 3:** Menstrual problems among students of BDU, ANRS, Ethiopia, 2010

Menstrual problem	Frequency (N=470)	Percent (%)
**Dysmenorrhea**		
Yes	400	85.1
No	70	14.9
**Grade of dysmenorrhea**		
Mild	190	47.5
Moderate	153	38.2
Severe	57	14.2
**PMS (at least one symptom)**		
Yes	342	72.8
No	128	27.2

The most common five psycho-behavioral premenstrual symptoms reported by the respondents were irritability (34.8%), fatigue (28.7%), depression (26.3%), anxiety/tension (23.7%) and social isolation/withdrawal. The five most common physical premenstrual symptoms reported by the respondents were breast tenderness (52.3%), bloating (33.9%), acne (26.0%), headache (19.0%), and joint or muscle pain (17.5%).

It was found that 28.5% of the respondents had participated in sexual intercourse. Out of the total respondents, those who smoked cigarette, drunk alcohol, and chewed khat were 2.3%, 21.9% and 3.6%, respectively. About 43.2% of the respondents participated in physical exercise.

The multivariate logistic regression which controls the undesirable effects of confounding variables was used by taking all eleven covariates (predictor variables) into account simultaneously for dysmenorrhea (which were significant at the bivariate analyses of dysmenorrhea in relation to each exploratory variable) and fifteen covariates for PMS (which were significant at the bivariate analyses of PMS in relation to each exploratory variable). The backward stepwise regression which controls the problem of multicollinearity was employed. During the multivariate analysis of dysmenorrhea in relation to all exploratory variables, only three of the most contributing factors remained to be statistically significant and independently associated with the presence of dysmenorrhea (at 0.05 level of significance) (Table 4). During the multivariate analysis of PMS in relation to all exploratory variables, only four of the most contributing factors remained to be statistically significant and independently associated with the presence of PMS (at 0.05 level of significance)(Table 5). Almost the same findings were also obtained by using the forward stepwise regression. The assessment made whether the required assumptions for the application of multiple logistic regression was fulfilled. It showed that the present parsimonious model adequately fits the data for dysmenorrhea and PMS as P- value from Hosmer and Lemeshow test was 0.782 and 0.823, respectively.

**Table 4:** Factors associated with Dysmenorrhea (Multivariate analysis), BDU, ANRS, Ethiopia, 2010

Explanatory variable	Dysmenorrhea (N=470)		COR	95%C.I.		AOR	95%C.I.		P-value
	Yes N(%)	No N(%)		Lower	Upper		Lower	Upper	
**Family history of dysmenorrhea**									
Yes	244(92.4)	20(7.6)	3.91	2.24	6.82	3.80	2.13	6.78	<0.001
No	156(75.7)	50(24.3)	1.00			1.00			
**Menstrual cycle length**									0.037*
< 21 days	16(88.9)	2(11.1)	0.24	0.03	1.80	0.36	0.04	3.03	0.348
21-35 days	316(82.7)	66(17.3)	0.14	0.03	0.59	0.16	0.04	0.71	0.015
> 35 days	68(97.1)	2(2.9)	1.00			1.00			
**Circumcision**									
Yes	189(90.4)	20(9.6)	2.24	1.29	3.90	1.84	1.001	3.386	0.050
No	211(80.8)	50(19.2)	1.00			1.00			

* For variables having more than two categories, the overall significance is given by their corresponding P-values

**Table 5:** Factors associated with PMS (Multivariate analysis), BDU, ANRS, Ethiopia, 2010

Explanatory variable	PMS (N=470)		COR	95%C.I		AOR	95%C.I.		P-Value
	Yes N(%)	No N(%)		Lower	Upper		Lower	Upper	
**Family history of PMS**									
Yes	210(85.4)	36(14.6)	4.07	2.61	6.33	4.19	2.60	6.74	<0.001
No	132(58.9)	92(41.1)	1.00			1.00			
**Campus**									
Poly	82(61.2)	52(38.8)	1.00			1.00			
Peda	260(77.4)	76(22.6)	2.17	1.41	3.34	2.11	1.30	3.45	0.003
**Menstrual regularity**									
Regular	167(66)	86(34)	1.00			1.00			
Irregular	175(80.6)	42(19.4)	2.146	1.402	3.284	1.87	1.17	2.99	0.008
**Educational status of mother**									0.016*
Illiterate	149(76)	47(24)	1.00			1.00			
Grade 1 to 6	73(72.3)	28(27.7)	0.82	0.48	1.42	0.78	0.43	1.42	0.422
Grade 7 - 12	70(82.4)	15(17.6)	1.47	0.77	2.81	1.42	0.70	2.90	0.330
Certificate and above	50(56.8)	38(43.2)	0.42	0.24	0.71	0.45	0.25	0.83	0.010

* For variables having more than two categories, the overall significance is given by their corresponding P-values.

## Discussion

The overall prevalence of dysmenorrhea (which is assumed to be primary dysmenorrhea since secondary dysmenorrhea is rare at this age) was 85.1% and according to the multidimensional scoring system of dysmenorrhea, the proportion of severe, moderate and mild dysmenorrheic respondents were 14.2%, 38.2%, and 47.5%, respectively. This was higher than a study conducted among secondary school students of Northern Ethiopia three years back by Desalegn and his colleagues, which was 72% [22]. It was also higher than the findings found in countries out of Africa and in Africa which was between 59.7% and 85% [11–22]. This might be due to the age range of the respondents which ranges between 17 and 24 by which the prevalence of dysmenorrhea is highest [6]. On the other hand, it might be related with the stress of the university students which has psychological impact that might induce dysmenorrhea. However; it was less than the prevalence found in a study conducted among university students of Turkey five years back which was 89.5% [15].

The prevalence of PMS (at least one symptom 1-7 days prior to menstruation in the last 12 months) was 72.80%. This was less than the prevalence found among Jima university students of Ethiopia, teacher training university students of Iran, university students of Thailand and university students of Nigeria which was 99.6%, 98.2%, 98% and 85.5%, respectively [26, 30–32]. However, it was higher than the prevalence found among university students of Saudi Arabia, college students of Japan and medical college students of India which was 35.6%, 43.3% and 60.7%, respectively [27–29]. This might be due to the difference in the study population, study period or diagnosis criteria of PMS. From the total number of respondents who had reported PMS which was 342(72.8%), only 76 (22.2%) of them had fulfilled the diagnostic criteria for PMDD (according to DSM-IV). Our diagnostic criteria for PMDD agreed with that of the study in Iran and Jima University and the prevalence was almost comparable with that of Jima University (27%) and Iran (16.9%) [26, 32].

Family history of dysmenorrhea was an important predictor for the presence of dysmenorrhea. It was found that those respondents who have family history of dysmenorrhea were 4 times more likely to have dysmenorrhea compared to those who do not have family history of dysmenorrhea (AOR=3.80, 95%CI: 2.13, 6.78). This was supported by a study in Malaysia secondary school students [17]. This implies that dysmenorrhea has genetic factor. It might have also a psychological impact. Daughters may react to menstruation similarly like their mothers and they may share the same attitude and taboos towards menses.

Menstrual cycle length had a direct effect on the presence of dysmenorrhea. Respondents who had menstrual cycle length of 21-35 days showed 84% reduction in the presence of dysmenorrhea compared to those respondents whose menstrual cycle length was > 35 days (AOR=0.16, 95%CI: 0.04, 0.71). This was consistent with the finding of a systemic review study in UK by which it was high for those long cycle length compared to the short one (AOR=1.46, 95% CI 1.01, 2.11) [25].

Circumcision had a positive impact on the presence of dysmenorrhea. The result found in this study after controlling other many variables indicated that those respondents who had been circumcised were 2 times more likely to have dysmenorrhea compared to those who had not been circumcised (AOR=1.84, 95%CI: 1.001, 3.386). This might be partly explained by the fact that circumcision causes multidimensional effect on the health of females. While talked more about the disadvantage of circumcision, those who had been circumcised before might feel and induced stress during menstruation. On the other hand, even if the degree was not assessed in this study, circumcision might have a physical scar that might be painful. This association was not assessed in many researches done before while it was known that circumcision had an impact on health in different aspects.

Having family history of PMS had a positive impact on the presence of PMS. Those students who had family history of PMS were 4.19 times more likely to have PMS compared to students who had no family history of PMS (AOR=4.19, 95%CI: 2.60, 6.74). This finding was consistent with study results among teacher training university students in Iran and university students in Saudi Arabia) [26, 27]. This shows that PMS has a genetic factor.

There was independent and very statistically significant linear association between campus and the odds of the presence of PMS among students of BDU. Those students of Peda campus were 2.11 times more likely to have PMS compared to students of Poly campus (AOR=2.11, 95%: 1.30, 3.45). Farther study is needed to conclude and recommend on this result.

Menstrual regularity was one of the factors associated with the presence of PMS. Those students who had irregular menstruation were 1.87 times more likely to have PMS compared to students who had regular menstruation (AOR=1.87, 95%CI: 1.17, 2.99). This finding was consistent with a study in Saudi Arabia (P < 0.001) and differs from a study of college students in Japan which had found no significant association between irregular menstruation and PMS [27, 28]. This might be due to the irregularity of menstruation which could fluctuate steroid hormones and might lead to PMS.

Educational status of the mother in general had the possibility of altering the presence of PMS in their daughters. An educational status of mothers with certificate and above is most likely to reduce PMS in their daughters by 55% than illiterate mothers (AOR=0.45, 95%CI: 0.25, 0.83). This result was different from a study conducted from university students of Saudi Arabia in which mother educational status was not significantly associated with PMS of their daughters (P=0.7). However; in this study, there was no evidence that students whose mothers had education up to grade 6 and students whose mothers had education from grade 7 to 12 had an effect on PMS compared to students whose mothers had no formal education which was (AOR=0.78, 95%CI: 0.43, 1.42) and (AOR=1.42, 95%CI: 0.70, 2.90), respectively. This might be due to the fact that educated mothers may discuss the problem with their daughters more freely than illiterate ones which in turn might help daughters to understand the problem better, and cope up with the problem better than those who had not enjoy free discussion with mothers.

## Conclusion

The prevalence of dysmenorrhea among students of BDU was very high. The prevalence of PMS among students of BDU was very high. Having family history of dysmenorrhea and being circumcised were the most positive contributing factors for the presence of dysmenorrhea whereas having menstrual cycle length of 21-35 days was the most contributing factor in reducing the presence of dysmenorrhea among students of BDU. Having irregular menstruation and family history of PMS were the most contributing factors in increasing the presence of PMS whereas educational status of mother being certified in certificate and beyond was the most contributing factor in reducing the presence of PMS among students of BDU. Health education, appropriate medical treatment and counseling, and recreational facilities should be accessible and persistently provided to the affected students by BDU. Even if the Ethiopian health policy is doing to eliminate the harmful traditional practices like female genital cutting (circumcision), maximum effort is needed to eliminate this activity by all levels. The effort of Ethiopian government to achieve the universal primary education among the Ethiopian nations should be encouraged and further steps that would enable females to join their college education should be applied.
